# Adverse events associated with poor neurological outcome during targeted temperature management and advanced critical care after out-of-hospital cardiac arrest

**DOI:** 10.1186/s13054-015-0991-9

**Published:** 2015-07-22

**Authors:** Young-Min Kim, Chun Song Youn, Soo Hyun Kim, Byung Kook Lee, In Soo Cho, Gyu Chong Cho, Kyung Woon Jeung, Sang Hoon Oh, Seung Pill Choi, Jong Hwan Shin, Kyoung-Chul Cha, Joo Suk Oh, Hyeon Woo Yim, Kyu Nam Park, on behalf of the Korean Hypothermia Network Investigators

**Affiliations:** Department of Emergency Medicine, College of Medicine, The Catholic University of Korea, 222 Banpo-daero, Seocho-gu, Seoul, 137-701 South Korea; Department of Emergency Medicine, College of Medicine, Chonnam National University, 160 Baekseo-ro, Dong-gu, Gwangju, 501-746 South Korea; Department of Emergency Medicine, KEPCO Medical Center, 308 Uicheon-ro, Dobong-gu, Seoul, 132-703 South Korea; Department of Emergency Medicine, College of Medicine, Hallym University, Kangdong Sacread Heart Hospital 150 Seongan-ro, Gangdong-gu, Seoul, 134-701 South Korea; Department of Emergency Medicine, Boramae Medical Center, Seoul National University, 20 Boramae-ro, Dongjak-gu, Seoul, 156-707 South Korea; Department of Emergency Medicine, Wonju College of Medicine, Yonsei University, 20 Ilsan-ro, Wonju, Gangwon-do, 220-701 South Korea; Department of Preventive Medicine, College of Medicine, The Catholic University of Korea, 222 Banpo-daero, Seocho-gu, Seoul, 137-701 South Korea

## Abstract

**Introduction:**

The aim of this study was to investigate the association of adverse events (AEs) during targeted temperature management (TTM) and other AEs and concomitant treatments during the advanced critical care period with poor neurological outcome at hospital discharge in adult out-of-hospital cardiac arrest (OHCA) patients.

**Methods:**

This was a retrospective study using Korean Hypothermia Network registry data of adult OHCA patients treated with TTM in 24 teaching hospitals throughout South Korea from 2007 to 2012. Demographic characteristics, resuscitation and post-resuscitation variables, AEs, and concomitant treatments during TTM and the advanced critical care were collected. The primary outcome was poor neurological outcome, defined as a cerebral performance category (CPC) score of 3–5 at hospital discharge. The AEs and concomitant treatments were individually entered into the best multivariable predictive model of poor neurological outcome to evaluate the associations between each variable and outcome.

**Results:**

A total of 930 patients, including 704 for whom a complete dataset of AEs and covariates was available for multivariable modeling, were included in the analysis; 476 of these patients exhibited poor neurological outcome [CPC 3 = 50 (7.1 %), CPC 4 = 214 (30.4 %), and CPC 5 = 212 (30.1 %)]. Common AEs included hyperglycemia (45.6 %), hypokalemia (31.3 %), arrhythmia (21.3 %) and hypotension (29 %) during cooling, and hypotension (21.6 %) during rewarming. Bleeding (5 %) during TTM was a rare AE. Common AEs during the advanced critical care included pneumonia (39.6 %), myoclonus (21.9 %), seizures (21.7 %) and hypoglycemia within 72 hours (23 %). After adjusting for independent predictors of outcome, cooling- and rewarming-related AEs were not significantly associated with poor neurological outcome. However, sepsis, myoclonus, seizure, hypoglycemia within 72 hours and anticonvulsant use during the advanced critical care were associated with poor neurological outcome [adjusted odds ratios (95 % confidence intervals) of 3.12 (1.40–6.97), 3.72 (1.93–7.16), 4.02 (2.04–7.91), 2.03 (1.09–3.78), and 1.69 (1.03–2.77), respectively]. Alternatively, neuromuscular blocker use was inversely associated with poor neurological outcome (0.48 [0.28–0.84]).

**Conclusions:**

Cooling- and rewarming-related AEs were not associated with poor neurological outcome at hospital discharge. Sepsis, myoclonus, seizure, hypoglycemia within 72 hours and anticonvulsant use during the advanced critical care period were associated with poor neurological outcome at hospital discharge in our study.

**Electronic supplementary material:**

The online version of this article (doi:10.1186/s13054-015-0991-9) contains supplementary material, which is available to authorized users.

## Introduction

Out-of-hospital cardiac arrest (OHCA) is an important public health problem in industrialized countries. In the United States, Europe, and South Korea, the estimated incidence of emergency medical services (EMS)-treated OHCA among individuals of any age is 5.7, 38, and 32.7 per 100,000 persons, respectively, and the median rate of survival to hospital discharge is 10.6 %, 10.7 %, and 3.7 %, respectively [1–3]. One of the worst complications of sudden cardiac arrest is hypoxic ischemic encephalopathy. Hypoxic ischemic encephalopathy after cardiac arrest results in death or disability in 50 % of patients who exhibit restoration of a pulse [4, 5]. Since positive results of a quasi-randomized controlled trial and a randomized controlled trial were reported in 2002, targeted temperature management (TTM) has been accepted as the only intervention shown in the last decade to improve outcomes for patients resuscitated from OHCA [6, 7]. Following these landmark studies, many observational studies have reported the effectiveness of TTM, and TTM is recommended as a component of routine post-cardiac arrest care in the international guidelines and a consensus report of five critical care societies [8–10]. Recently, well-performed, large randomized controlled trials also highlighted that targeting a temperature of 36 °C gives equal results as targeting a temperature of 33 °C [11–13].

Induced hypothermia may cause various physiological changes as well as adverse events (AEs) [14, 15]. Although AEs commonly occur during TTM and the advanced critical care period, the incidence of most AEs is not significantly different between induced hypothermia and normothermia treatment [16, 17]. Although some investigators have reported an association between AEs recorded during critical care and mortality, few studies have investigated the relationship between AEs and neurological outcomes in patients with return of spontaneous circulation (ROSC) after OHCA [18–20]. Furthermore, most previous studies have been performed in Western countries, which have relatively high bystander cardiopulmonary resuscitation (CPR) rates and well-established EMS systems and where the withdrawal of life-sustaining treatment (WLST) is permitted according to the legislation.

The aim of this study was to investigate the association of cooling-related AEs, rewarming-related AEs, other AEs, and concomitant treatments during the advanced critical care period with poor neurological outcome at the time of hospital discharge in adult OHCA patients who received TTM in South Korea; the baseline characteristics of South Korean adult OHCA patients are different from those of adult OHCA patients in Western countries, and WLST based on prognostication is not yet permitted in South Korea.

## Materials and methods

### Patients

This was a multicenter, retrospective, registry-based study. This study used the Korean Hypothermia Network (KORHN) registry data. The KORHN, a multicenter clinical research consortium for TTM in South Korea, was organized in 2011 and performed the multicenter retrospective registry project in 2012. The KORHN investigators retrospectively collected data from OHCA patients treated with TTM and advanced critical care at 24 teaching hospitals throughout South Korea from 2007 to 2012. The 24 centers used their own protocol for TTM, but the protocols of most centers included the same parameters of TTM (target temperature of 33 °C, 24-hour maintenance, and controlled normothermia for 72 hours after ROSC). The institutional review board of each institution approved the study protocol before data collection. The name of ethics bodies and site principal investigators are listed in the Acknowledgements section. The institutional review boards waived the requirement to obtain informed consent due to the retrospective nature of the study.

Adult (≥18 years) OHCA patients treated with TTM after ROSC were included in the study. Traumatic cardiac arrest patients were excluded. The case report form, standard definitions of 87 variables, and an investigator manual were developed based on a literature review and a consensus of the study investigators (see Additional file 1). The definitions and time points of the variables followed the Utstein recommendations [21, 22]. The registry data were collected from reviews of medical charts or electronic medical records. The data collected from each hospital were verified for completeness by the site principal investigator and were recorded in a web-based data registration system by the site clinical research coordinator. A data manager and three clinical research associates monitored and regularly reviewed data quality. The site principal investigators or site clinical research coordinators were contacted using the query function in the system or directly by phone to clarify data.

### Data collection

We collected data, including demographic characteristics, resuscitation variables, post-resuscitation variables, and AEs and concomitant treatments during TTM and the advanced critical care period. The detailed definitions of the variables and other items included in the registry are described in an Additional file (see Additional file 2) and were reported in an article describing the KORHN registry project [23]. The AEs were divided into cooling-related, rewarming-related, and other AEs during the advanced critical care period. The considered cooling-related and rewarming-related AEs were those which were commonly reported in the literature and were typically attributed to TTM [14, 15]. The terminology of advanced critical care was derived from the American Heart Association adult immediate post-cardiac arrest care algorithm [8]. The advanced critical care period was defined as days 1–7 after ROSC, which was the definition used in previous AE studies [11, 24]. We have included the period as a figure form in a practical manual of post-cardiac arrest care for hospital providers developed by the KORHN. We added the figure in an Additional file (see Figure S1 in Additional file 3).

Overcooling (<32 °C), bradycardia (<40 beats/min), tachyarrhythmia, hypokalemia (<3.0 mEq/L), hyperglycemia (>180 mg/dL), bleeding, and hypotension were collected as cooling-related AEs. Rebound hyperthermia (>38 °C within 24 hours of the cessation of cooling), arrhythmia, hyperkalemia (>5.0 mEq/L), hypoglycemia (<80 mg/dL), bleeding, and hypotension were collected as rewarming-related AEs. Tachyarrhythmia during cooling was defined as newly developed tachyarrhythmia during cooling except for sinus tachycardia. Bleeding was defined as any site of bleeding associated with cooling and rewarming. Hypotension was defined as systolic blood pressure (SBP) <90 mmHg or mean arterial pressure (MAP) <60 mmHg for at least 30 min or requiring supportive measures (fluid loading and treatment with a vasopressor or inotropic drug or both types of drugs) to maintain the SBP >90 mmHg or the MAP >60 mmHg during cooling and rewarming.

Sepsis, pneumonia, myoclonus and seizure occurring during the advanced critical care period that could not be clearly categorized as typical cooling-related or rewarming-related AEs were categorized as other AEs. All hypoglycemic events (defined as <80 mg/dL within 72 hours after ROSC and collected in the registry) occurring during the advanced critical care period were categorized as other AEs. The use of analgesics, sedatives, neuromuscular blockers (NMBs), anticonvulsants and insulin during TTM and the advanced critical care period was categorized as concomitant treatment. Sepsis was defined as a clinical syndrome defined by the presence of both documented or suspected infection and systemic inflammatory response syndrome; these cases were categorized as sepsis, severe sepsis, or septic shock [25]. Pneumonia was diagnosed based on the following four clinical criteria without microbiological confirmation: new or progressive consolidation on a chest radiograph, fever, leukocytosis, and the presence of purulent tracheobronchial secretions [19]. Seizure was diagnosed based on either clinically involuntary movements with epileptiform discharges on electroencephalography (EEG) or epileptiform discharges alone on EEG. Myoclonus was defined as clinically involuntary movement without epileptiform discharges on EEG.

### Outcome measures

The goal of our study was to examine the association between AEs and poor neurological outcome at hospital discharge. Poor neurological outcome was defined as a cerebral performance category (CPC) scale score of 3–5. Based on the traditional criteria, CPC 3 indicates severe cerebral disability (conscious but disabled and dependent), CPC 4 indicates coma or a vegetative state (unconscious), and CPC 5 indicates brain death or death [26]. The CPC scores at hospital discharge, which were recorded in the medical charts and the electronic medical records by attending physicians or independent neurologists, were collected. If the score was not recorded, site investigators abstracted these data from the medical records by consensus.

### Statistical analysis

Continuous variables are expressed as the means and standard deviations or the medians and interquartile ranges according to normal distributions, and categorical variables are expressed as numbers and percentages. We used the Wilcoxon rank sum test or the *t* test and the chi-squared test as appropriate for univariate comparisons of the baseline characteristics between patients with good and poor neurological outcome. First, we determined the clinical variables that were independently associated with functional outcome. Second, we determined the association of cooling-related AEs, rewarming-related AEs, and other AEs during the advanced critical care period with functional outcome, adjusting for the clinical variables identified during the first step.

A multivariate analysis of the relationship between functional outcome and the baseline characteristics was performed using a stepwise logistical regression method, which utilizes a combination of forward and backward selection for multivariable predictive modeling. Variables displaying *p* ≤0.15 based on univariate analysis were considered as candidates for inclusion in the multivariable predictive model. Continuous variables entered into the model included age (in years), time from arrest to ROSC (in minutes), time from arrest to initiation of cooling (in minutes), time from arrest to 34 °C (in minutes), and rewarming time (in minutes). Dichotomous variables included sex (male as reference), previously unhealthy (absence of the listed diseases as reference), underlying disease (absence of disease as reference), unwitnessed arrest (witnessed as reference), no bystander CPR (bystander CPR as reference), non-shockable initial rhythm (ventricular fibrillation/pulseless ventricular tachycardia as reference), non-cardiac cause of arrest (cardiac cause of arrest as reference), initial Glasgow coma scale (GCS) score (<5 as reference), initial presence of the pupillary light reflex (absence of the pupillary light reflex as reference), ST-segment elevation myocardial infarction (STEMI) (non-STEMI as reference), cardiogenic shock (no cardiogenic shock as reference), and hospital interventions (no intervention as reference). The results are presented as the mean or median differences with *p* values or odds ratios (ORs) with 95 % confidence intervals (CIs). The goodness-of-fit of the final model was assessed using the Hosmer-Lemeshow test with chi-square analysis and max-rescaled R [2]. To assess the predictiveness of the final model, we calculated the area under the receiver operating characteristic curve (AUC). As a sensitivity analysis, we assessed the adequacy of the final model including all available data from 930 subjects.

The AEs and the concomitant treatments were individually entered into the multivariable predictive model adjusting for the confounders of outcome to evaluate their association with functional outcome. The results are presented as adjusted ORs with 95 % CIs. For exploratory analyses, we performed univariate logistic regression analyses to evaluate the association between each AE and the clinical variables. Additionally, we performed a sensitivity analysis using the data from the patients who survived until sedation was withdrawn (approximately 3 days after ROSC) to focus on the association between the AEs and functional outcome among these patients. The analyses were conducted using SAS version 9.2 (SAS Institute Inc., Cary, NC, USA). Two-tailed tests of significance were used, and *p* <0.05 was considered to be statistically significant in the analyses.

## Results

### Baseline patient characteristics and univariate comparisons of the characteristics

**Fig. 1:**
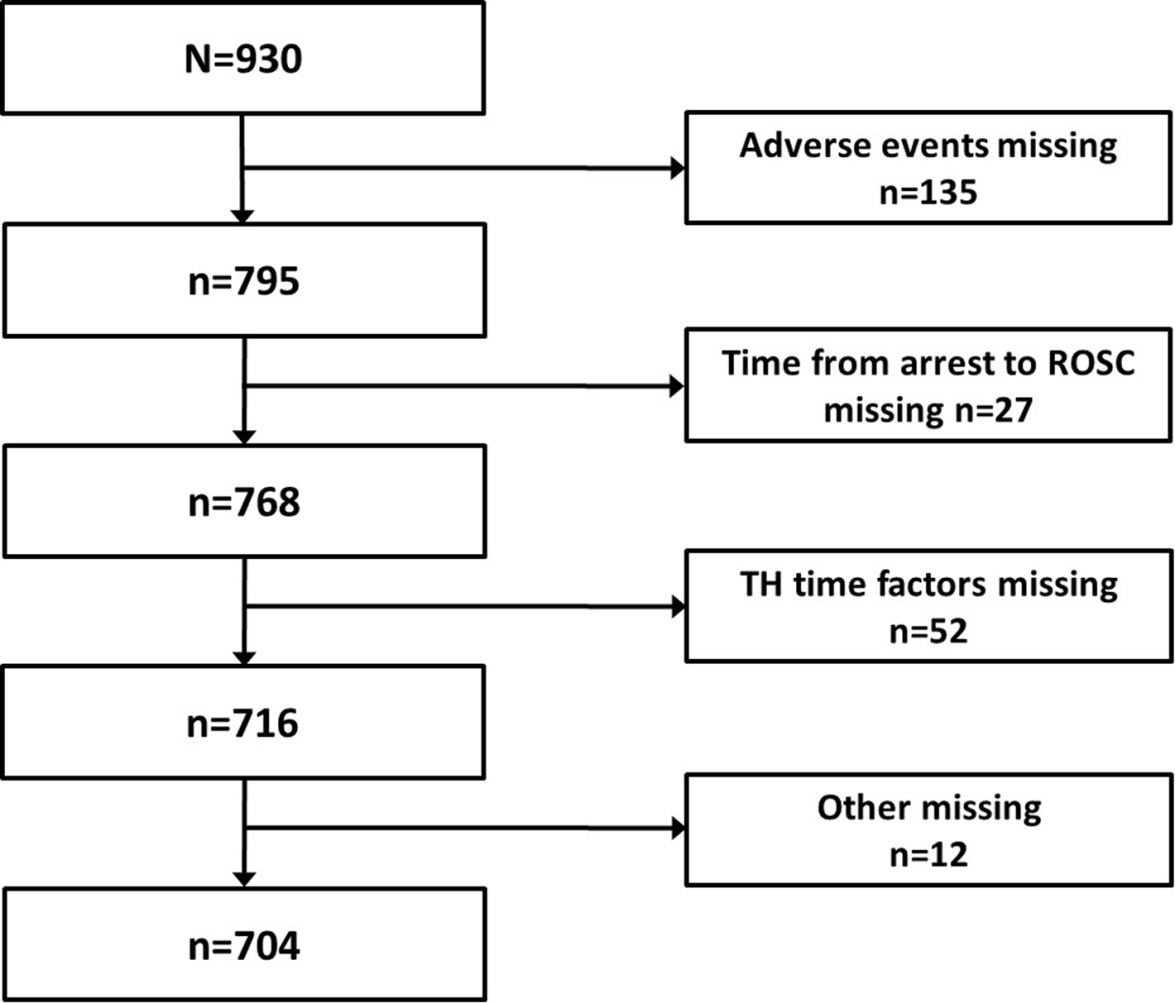
Flow diagram of the patient selection process

A total of 930 patients from 24 hospitals were included in the registry. The geographic distribution and the number of patients enrolled in the 24 participating centers were reported as a figure form in an article describing the KORHN registry project [23]. We added the figure in an Additional file (see Figure S2 in Additional file 3). Among these patients, 226 patients for whom data were missing were excluded; as a result, 704 patients were included in the analysis (Fig. 1). Table 1 provides the baseline characteristics, including demographics, resuscitation variables, and post-resuscitation variables, of the included patients and the univariate comparisons of these characteristics between those with good and poor neurological outcome. Of the 228 (32.4 %) patients with good neurological outcome, 193 (27.4 %) and 35 (4.9 %) patients were discharged with CPC 1 and 2, respectively. Among the 476 (67.6 %) patients with poor neurological outcome, 50 (7.1 %), 214 (30.4 %), and 212 (30.1 %) exhibited CPC of 3, 4, and 5, respectively. In our study, surviving OHCA patients were discharged after an average of 14–15 days at the hospital.

**Table 1:** Baseline patient characteristics

Patient characteristics	Good neurological outcome (CPC 1 and 2) n = 228 (32.4)	Poor neurological outcome (CPC 3–5) n = 476 (67.6)	*p*
Background variables			
Age, years	50 (40–60)	59 (47–71)	<.001
Male	177 (77.6)	323 (67.9)	0.008
Previously healthy^a^	130 (57.0)	178 (37.4)	<.001
Myocardial infarction	13 (5.7)	26 (5.5)	0.897
Angina	19 (8.3)	29 (6.1)	0.270
Congestive heart failure	5 (2.2)	19 (4.0)	0.219
Hypertension	57 (25.0)	172 (36.1)	0.003
Diabetes mellitus	29 (12.7)	116 (24.4)	<.001
Pulmonary disease	4 (1.8)	35 (7.4)	0.002
Stroke	4 (1.8)	27 (5.7)	0.018
Other neurological disease	7 (3.1)	35 (7.4)	0.025
Renal disease	4 (1.8)	37 (7.8)	0.001
Liver cirrhosis	1 (0.4)	9 (1.9)	0.180
Malignancy	5 (2.2)	14 (2.9)	0.567
Resuscitation variables			
Witnessed arrest	182 (79.8)	290 (60.9)	<.001
Bystander CPR	85 (37.3)	121 (25.4)	0.001
First monitored rhythm			<.001
VF/pulseless VT	148 (64.9)	89 (18.7)	
Pulseless electrical activity	28 (12.3)	77 (16.2)	
Asystole	41 (17.9)	299 (62.8)	
Unknown	11 (4.8)	11 (2.3)	
Cardiac cause of arrest	201 (88.2)	232 (48.7)	<.001
Time from arrest to ROSC, min	24.5 (16.5–34.5)	33 (25–45)	<.001
Post-resuscitation variables			
Initial Glasgow coma scale ≥5	76 (33.3)	23 (4.8)	<.001
Initial presence of pupillary light reflex	178 (78.1)	132 (27.7)	<.001
ST-elevation myocardial infarction	36 (15.8)	26 (5.5)	<.001
Cardiogenic shock^b^	64 (28.1)	127 (26.7)	0.698
Thrombolysis	2 (0.9)	3 (0.6)	0.661
Coronary angiography	152 (66.7)	50 (10.5)	<.001
Percutaneous coronary intervention	44 (19.3)	25 (5.3)	<.001
Coronary artery bypass graft	7 (3.1)	0 (0.0)	<.001
Intra-aortic balloon pump	8 (3.5)	11 (2.3)	0.359
Extracorporeal membrane oxygenation	4 (1.8)	10 (2.1)	1.000
Continuous renal replacement therapy	4 (1.8)	49 (10.3)	<.001
Time from arrest to initiation of cooling, min	142 (81.5–247.5)	140 (80–240)	0.629
Time from arrest to 34 °C, min	385 (257.5–570)	307 (200–2454.5)	<.001
Rewarming time, min	666 (420–780)	720 (480–990)	0.002

Data are presented as medians (interquartile range) or counts (percentage)

*CPC* cerebral performance category, *CPR* cardiopulmonary resuscitation, *VF* ventricular fibrillation, *VT* ventricular tachycardia, *ROSC* return of spontaneous circulation

^a^Absence of the listed diseases

^b^Hypotension (SBP <90 mmHg or MAP <60 mmHg for at least 30 min or the need for supportive measures (fluid loading and vasopressors or inotropic or both) to maintain a SBP >90 mmHg or MAP >60 mmHg) and end-organ hypoperfusion (cool extremities)

### Multivariable predictive modeling of poor neurological outcome

The best multivariable model for predicting poor neurological outcome at the time of hospital discharge included nine independent variables (Table 2). The final model showed the following characteristics: a Hosmer-Lemeshow test: chi-square = 13.6, *p* = 0.093; max-rescaled R [2] = 0.659; and AUC = 0.929. Based on a sensitivity analysis of 882 subjects for whom data for the variables were available, the final model also performed well (Hosmer-Lemeshow test: chi-square = 13.6, *p* = 0.714; max-rescaled R [2] = 0.655; and AUC = 0.933).

**Table 2:** Multivariable predictive model for poor neurological outcomes at the time of hospital discharge

Variables	OR	(95 % CI)	*p*
Continuous renal replacement therapy^a^	5.66	(1.85–21.97)	0.005
Non-shockable initial rhythm^b^	2.97	(1.72–5.16)	0.009
Non-cardiac cause of arrest^c^	2.73	(1.47–5.17)	0.002
Previously unhealthy^d^	2.07	(1.20–3.60)	0.001
Time from arrest to ROSC^e^	1.06	(1.04–1.08)	<.001
Age^f^	1.03	(1.01–1.05)	0.001
Initial Glasgow coma scale ≥5^g^	0.29	(0.14–0.58)	0.001
Initial presence of pupillary light reflex^h^	0.28	(0.17–0.46)	<.001
Coronary angiography^i^	0.13	(0.14–0.58)	<.001

*OR* odds ratio, *95 % CI* 95 % confidence interval, *ROSC* return of spontaneous circulation

^a^No continuous renal replacement therapy as reference

^b^VF/pulseless VT as reference

^c^Cardiac cause of arrest as reference

^d^Absence of the listed diseases as reference

^e^By minute

^f^By year

^g^ < 5 as reference

^h^Absence of pupillary light reflex as reference

^i^No coronary angiography as reference

### Adverse events associated with poor neurological outcome

The frequencies of TTM-related (cooling- and rewarming-related), and other AEs during the advanced critical care period and concomitant treatments during TTM and the advanced critical care period, along with corresponding odds ratios for poor neurological outcome, are presented in Table 3. Table 4 shows the factors associated with selected AEs based on univariate analysis.

**Table 3:** Adverse events and concomitant treatments with corresponding odds ratios for poor neurological outcomes at the time of hospital discharge

Category	AEs and concomitant treatments	Total N (%)	CPC 1 and 2 n (%)	CPC 3–5 n (%)	OR (95 % CI)	*p*	Adjusted OR (95 % CI)	*p*
		704 (100)	228(32.4)	476 (67.6)				
Cooling -related AEs	Overcooling^a^	128 (18.2)	23 (10.1)	105 (22.1)	2.52 (1.56–4.09)	<.001	1.16 (0.58–2.31)	0.681
	Bradycardia^b^	83 (11.8)	35 (15.4)	48 (10.1)	0.62 (0.39–0.99)	0.044	0.75 (0.37–1.53)	0.429
	Tachyarrhythmia	67 (9.5)	24 (10.5)	43 (9.0)	0.84 (0.50–1.43)	0.528	0.72 (0.33–1.55)	0.398
	Hypokalemia^c^	220 (31.3)	65 (28.5)	155 (32.6)	1.21 (0.86–1.71)	0.278	1.21 (0.71–2.04)	0.486
	Hyperglycemia^d^	321 (45.6)	81 (35.5)	240 (50.4)	1.85 (1.33–2.56)	<.001	1.37 (0.84–2.24)	0.210
	Bleeding	21 (3.0)	6 (2.6)	15 (3.2)	1.20 (0.46–3.15)	0.705	0.89 (0.21–3.71)	0.872
	Hypotension^e^	204 (29.0)	54 (23.7)	150 (31.5)	1.48 (1.03–2.13)	0.033	1.03 (0.59–1.79)	0.926
Rewarming -related AEs	Rebound hyperthermia^f^	85 (12.1)	29 (12.7)	56 (11.8)	0.92 (0.57–1.48)	0.716	1.33 (0.61–2.92)	0.473
	Arrhythmia	42 (6.0)	10 (4.4)	32 (6.7)	1.57 (0.76–3.25)	0.224	1.70 (0.61–4.69)	0.308
	Hyperkalemia^g^	48 (6.8)	7 (3.1)	41 (8.6)	2.96 (1.31–6.74)	0.009	1.09 (0.37–3.19)	0.881
	Hypoglycemia^h^	70 (9.9)	22 (9.7)	48 (10.7)	1.05 (0.62–1.79)	0.858	1.14 (0.50–2.57)	0.762
	Bleeding	14 (2.0)	3 (1.3)	11 (2.3)	1.77 (0.49–6.41)	0.383	1.05 (0.18–6.06)	0.956
	Hypotension^e^	152 (21.6)	36 (15.8)	116 (24.4)	1.72 (1.14–2.60)	0.01	0.99 (0.54–1.83)	0.984
Other AEs during advanced critical care Concomitant treatments	Pneumonia^i^	279 (39.6)	70 (30.7)	209 (43.9)	1.77 (1.26–2.47)	<.001	1.05 (0.63–1.74)	0.852
	Sepsis	100 (14.2)	17 (7.5)	83 (17.4)	2.62 (1.52–4.53)	<.001	3.12 (1.40–6.97)	0.005
	Myoclonus^j^	154 (21.9)	34 (14.9)	120 (25.2)	1.92 (1.27–2.92)	0.002	3.72 (1.93–7.16)	<.001
	Seizure^k^	153 (21.7)	24 (10.5)	129 (27.1)	3.16 (1.98–5.05)	<.001	4.02 (2.04–7.91)	<.001
	Hypoglycemia^l^	162 (23.0)	36 (15.8)	126 (26.5)	1.92 (1.27–2.89)	0.002	2.03 (1.09–3.78)	0.025
	Anticonvulsants	287 (40.8)	71 (31.1)	216 (45.4)	1.84 (1.32–2.56)	<.001	1.69 (1.03–2.77)	0.039
	Insulin	460 (65.3)	136(59.7)	324 (68.1)	1.44 (1.04–2.00)	0.028	0.98 (0.60–1.62)	0.944
	Analgesics	323 (45.9)	122 (53.5)	201 (42.2)	0.64 (0.46–0.87)	0.005	0.75 (0.46–1.21)	0.239
	Sedatives	657 (93.3)	222 (97.4)	435 (91.4)	0.29 (0.12–0.69)	0.005	0.92 (0.29–2.96)	0.888
	Neuromuscular blockers	475 (67.5)	174 (76.3)	301 (63.2)	0.53 (0.37–0.76)	0.001	0.48 (0.28–0.84)	0.009

*AEs* adverse events, *CPC* cerebral performance category, *OR* odds ratio, *95 % CI* 95 % confidence interval

^a^ < 32 °C

^b^Heart rate <40 bpm

^c^ ≤ 3.0 mEq/L

^d^ ≥ 180 mg/Dl

^e^SBP <90 mmHg or MAP <60 mmHg for at least 30 min or the need for supportive measures (fluid loading and vasopressors or inotropic or both) to maintain a SBP >90 mmHg or MAP >60 mmHg

^f^ > 38 °C within 24 hours of the cessation of cooling

^g^ ≥ 5.0 mEq/L

^h^ ≤ 80 mg/dL

^i^Four requirements: 1) new or progressive consolidation on the chest radiograph, 2) fever, 3) leukocytosis, and 4) the presence of purulent tracheobronchial secretions

^j^Clinically involuntary movement without epileptiform discharge on EEG

^k^Either clinically involuntary movement with epileptiform discharge on electroencephalogram (EEG) or epileptiform discharge only on EEG

^l^ ≤ 80 mg/dL within 72 hours after ROSC

**Table 4:** Factors associated with selected adverse events in univariate analyses

Adverse event	Factors	OR	(95 % CI)	*p*
Bleeding	Intra-aortic balloon pump	5.40	(1.47–19.83)	0.011
	Continuous renal replacement therapy	3.13	(1.13–8.65)	0.028
	Cardiogenic shock^a^	2.39	(1.09–5.26)	0.031
Pneumonia^b^	Ice bag only	3.00	(1.79–5.02)	<.001
	Malignancy	2.68	(1.04–6.90)	0.041
	Neurological disease	2.36	(1.25–4.46)	0.008
	Non-shockable initial rhythm	1.58	(1.14–2.20)	0.006
	No bystander cardiopulmonary resuscitation	1.45	(1.03–2.03)	0.033
	Non-cardiac cause of arrest	1.44	(1.06–1.96)	0.021
	Initial presence of pupillary light reflex	0.57	(0.42–0.78)	<.001
	ST-elevation myocardial infarction	0.55	(0.31–0.98)	0.042
	Coronary angiography	0.51	(0.36–0.73)	<.001
	Angina pectoris	0.49	(0.25–0.95)	0.035
	Percutaneous coronary intervention	0.47	(0.26– 0.82)	0.008
	Initial Glasgow coma scale (≥5)	0.44	(0.27– 0.72)	0.001
Sepsis	Intra-aortic balloon pump	3.71	(1.43–9.67)	0.007
	Ice bag only	2.17	(1.20–3.94)	0.010
	Unwitnessed arrest	2.11	(1.37–3.24)	0.001
	No bystander cardiopulmonary resuscitation	1.78	(1.06–2.99)	0.030
	Cardiogenic shock^a^	1.72	(1.10–2.68)	0.017
	Non-cardiac cause of arrest	1.65	(1.08–2.53)	0.021
	Coronary angiography	0.58	(0.35–0.98)	0.040
Myoclonus^c^	Ice bag only	2.40	(1.42–4.05)	0.001
	Hydrogel pad	1.58	(1.05–2.38)	0.027
	ST-elevation myocardial infarction	0.36	(0.15–0.85)	0.019
	Cooling garment	0.06	(0.01–0.45)	0.006
Seizure^d^	Congestive heart failure	2.68	(1.17–6.17)	0.020
	Non-shockable initial rhythm	1.51	(1.01–2.25)	0.043
	Endovascular cooling	0.64	(0.42–0.97)	0.035
	Coronary angiography	0.54	(0.35–0.83)	0.005
	Initial Glasgow coma scale ≥ 5	0.32	(0.16–0.65)	0.002
Hypoglycemia^e^	Continuous renal replacement therapy	2.59	(1.45–4.62)	0.001
	Renal disease	2.53	(1.32–4.84)	0.005
	Non-shockable initial rhythm	2.06	(1.37–3.11)	<.001
	Coronary angiography	0.65	(0.43–0.98)	0.039
	Hydrogel pad	0.55	(0.34–0.88)	0.013
	Percutaneous coronary intervention	0.47	(0.23–0.98)	0.042
	Cooling garment	0.25	(0.09–0.70)	0.008

*OR* odds ratio, *95 % CI* 95 % confidence interval

^a^Hypotension (SBP <90 mmHg or MAP <60 mmHg for at least 30 min or the need for supportive measures (fluid loading and vasopressors or inotropic or both) to maintain a SBP >90 mmHg or MAP >60 mmHg) and end-organ hypoperfusion (cool extremities)

^b^Four requirements: 1) new or progressive consolidation on the chest radiograph, 2) fever, 3) leukocytosis, 4) presence of purulent tracheobronchial secretions

^c^Clinically involuntary movement without epileptiform discharge on EEG

^d^Either clinically involuntary movement with epileptiform discharge on EEG or epileptiform discharge only on EEG

^e^ ≤ 80 mg/dL within 72 hours after ROSC

#### TTM-related AEs

Overcooling, hyperglycemia, and hypotension during the cooling period and hyperkalemia and hypotension during the rewarming period were more frequently observed in patients with poor neurological outcome than in patients with good neurological outcome. However, after adjusting for independent predictors of poor neurological outcome, these AEs were not significantly associated with poor neurological outcome.

Newly developed arrhythmia during TTM was observed in 192 (27.3 %) patients and most frequently developed during the cooling period. Bradycardia was more frequently observed in patients with good neurological outcome based on univariate analysis. However, after adjusting for independent predictors of poor neurological outcome, bradycardia was not significantly associated with poor neurological outcome.

Bleeding developed in 35 patients (5 %) during TTM, and 17 patients (2.4 %) required a transfusion. Among these patients, cooling was stopped due to severe bleeding for only 1 patient (0.1 %). Based on univariate analysis, intra-aortic balloon pump (IABP) use, continuous renal replacement therapy (CRRT), and cardiogenic shock were associated with bleeding. There was no significant difference in the incidence of bleeding between patients with good and poor neurological outcome at hospital discharge.

#### Other AEs during the advanced critical care period

Pneumonia and sepsis were associated with poor neurological outcome based on univariate analysis. After adjusting for independent predictors of poor neurological outcome, sepsis, but not pneumonia, was significantly associated with poor neurological outcome.

Myoclonus, seizure, and hypoglycemia within 72 hours after ROSC were associated with poor neurological outcome based on univariate analysis. After adjusting for the independent predictors of poor neurological outcome, myoclonus, seizure, and hypoglycemia within 72 hours after ROSC remained significantly associated with poor neurological outcome.

#### Concomitant treatment during TTM and the advanced critical care period

Patients with poor neurological outcome were more frequently treated with anticonvulsants or insulin than those with good neurological outcome. After adjusting for independent predictors of poor neurological outcome, anticonvulsant use, but not insulin use, was significantly associated with poor neurological outcome. Alternatively, the use of analgesics, sedatives and NMBs was inversely associated with poor neurological outcome based on univariate analysis. After adjusting for independent predictors of poor neurological outcome, only NMB use was inversely associated with poor neurological outcome.

#### Sensitivity analysis of the patients who survived after sedation was withdrawn

A total of 32 patients (4.5 %) died within 3 days after ROSC. Based on sensitivity analysis of the remaining 672 patients, the best multivariable model for predicting poor neurological outcome included the same nine independent variables described above. This model displayed the following characteristics: Hosmer-Lemeshow test: chi-square = 7.5, *p* = 0.487; max-rescaled R [2] = 0.668; and AUC = 0.930. The same four AEs and one concomitant treatment were significantly associated with poor neurological outcome at hospital discharge based on univariate and multivariate analyses (see Additional file 4).

## Discussion

In this multicenter, registry-based study of adult OHCA patients treated with TTM, cooling-related or rewarming-related AEs were not significantly associated with poor neurological outcome at hospital discharge after adjusting for significant predictors of poor neurological outcome. However, sepsis, myoclonus, seizure, hypoglycemia within 72 hours after ROSC and anticonvulsant use during the advanced critical care period were significantly associated with poor neurological outcome at hospital discharge. Based on sensitivity analysis of the 672 patients who survived after sedation was withdrawn, the results were consistent with the findings of the primary analysis.

To the best of our knowledge, this is the largest observational study to evaluate the association of AEs occurring during TTM and the advanced critical care period with neurological outcome at hospital discharge in adult OHCA patients treated with TTM. Although we were only able to investigate neurological outcome at hospital discharge, the outcomes reported in our study may represent the natural course of OHCA patients treated with TTM in the hospitals because WLST based on prognostication was not applied to these patients.

The results of neurological outcome among our survivors were quite different from those observed in previous studies performed in Western countries [11, 18, 19]. Compared to the results of prospective observational studies by Nielsen et al. [18, 19], in which only a few patients (1 %) exhibited CPC 4, 214 patients (30.4 %) exhibited CPC 4 in our study. In the Target Temperature Management trial, only 19 (4 %) patients at hospital discharge and 6 (1 %) patients at 6 months had CPC 4 [11]. These differences may result from the difference in baseline patients’ characteristics, especially certain resuscitation-related variables, lower rate of WLST in our study population, and difference in end points between two studies (at 6 months versus at hospital discharge). Because prehospital chains are relatively weak in South Korea, resuscitation variables such as witnessed arrest, bystander CPR, and initial rhythm were different in our study compared to previous studies performed in Western countries. In prospective observational studies from Europe and North America, the proportions of witnessed arrest, bystander CPR, and initial shockable rhythm were higher than those in our study [11, 18, 19]. In contrast to the rate of WLST in Western countries, where WLST is the most common reason for death among cardiac arrest survivors [5, 27], the rate of WLST is very low in South Korea due to local cultural factors. Regardless of neurological status or the results of prognostication tests, most family members choose to continue care for comatose survivors resuscitated from cardiac arrest. Although the Korean Medical Association recommended consensus guidelines in 2009, WLST remains a matter of debate, and WLST for post-cardiac arrest patients based on prognostication is not legally permitted in South Korea. This circumstance provides an excellent opportunity to collect unbiased data on the association between prognostic factors including AEs and ultimate neurological outcomes in the modern intensive care setting. One of the most valuable results of our study is the generation of a multivariable predictive model of poor neurological outcome at the time of hospital discharge. Because this model was fitted to data in which WLST was not performed, our results address a critical limitation of most modern studies from Europe and North America.

*Bleeding during TTM* was rare in our study, and its incidence was similar to that of most previous reports [7, 11, 16–20]. Bleeding during TTM was more frequently observed in patients receiving an invasive intervention, such as IABP, extracorporeal membrane oxygenation (ECMO), and cardiogenic shock, whereas thrombolysis and the use of an endovascular device, coronary angiography, or percutaneous coronary intervention (PCI) were not associated with an increased risk of bleeding based on univariate analysis. These results do not correspond to the findings of the prospective study by Nielsen et al., in which thrombolysis and the use of an endovascular device were associated with an increased risk of bleeding based on univariate analysis [19]. However, bleeding was not significantly associated with the outcome in either study. These results are consistent with the conclusion of a recent systematic review and meta-analysis of the risk of bleeding [28].

The incidence of *newly developed arrhythmia during TTM* was slightly lower than that observed in previous studies [11, 19, 29, 30]. Because we only retrospectively collected the arrhythmia data during the TTM period, the incidence observed in this study could be lower than that observed by others. Recently, bradycardia during TTM has been suggested to be a predictor of favorable outcome [29, 30]. In our study, bradycardia during the cooling period more frequently developed in patients with good neurological outcome based on univariate analysis. However, after adjusting for independent predictors of poor neurological outcome, bradycardia was not significantly associated with poor neurological outcome at hospital discharge.

*Hypotension during TTM* was common but was not significantly associated with poor neurological outcome at hospital discharge after adjusting for independent predictors in our study. This finding is consistent with the results of a previous study showing that MAP time integral during TTM were not significantly associated with functional outcome at hospital discharge [31]. However, the impact of hypotension during TTM on functional outcome is controversial. Many studies showed that early hypotension (at the time of admission or during the first 6 hours after ROSC) and need for persisted vasopressor support during TTM was significantly associated with poor outcome [32–34]. Although hypotension during TTM was not associated with poor neurological outcome in our study, any conclusions based on our results should be made with caution because the cause of hypotension is multifactorial and because persistent or severe hypotension during TTM is generally rare.

*Overcooling* was one of the most concerning AEs observed during the cooling period [35, 36]. Although overcooling during the cooling period was associated with poor neurological outcome based on univariate analysis, no such association was observed based on multivariate analysis in our study. The incidence (12 %) of *rebound hyperthermia* in our study was relatively lower than that in previous reports of post-hypothermia fever [37–40]. This discrepancy may be related to the differences in the definitions of temperature level, observation period and different populations between the compared studies. In our study, which defined post-hypothermia fever (>38 °C) within 24 hours of the cessation of cooling, rebound hyperthermia was not associated with poor neurological outcome based on either univariate or multivariate analysis. The influence of post-hypothermia fever on functional outcome is controversial [41, 42]. Therefore, further research on these temperature control-related AEs is necessary.

The incidence (39.6 %) of *pneumonia during the advanced critical care period* was similar to that described in most previous reports, including studies of cardiac arrest patients treated with TTM [7, 17, 18]. However, in some studies, the pneumonia incidence was higher than our result [11, 19, 43]. Differences in definitions, the use of prophylactic antibiotics, and the rate of WLST between studies may explain these discrepancies in the pneumonia incidence. In a recent report, prophylactic antibiotic use was associated with a lower incidence of pneumonia but did not appear to influence mortality or functional outcome in cardiac arrest survivors treated with TTM [44]. Because most participating hospitals in our registry used prophylactic antibiotics in their protocols, the incidence of pneumonia in our study may be lower than that in other studies [11, 19, 43]. However, the impact of prophylactic antibiotic use on functional outcome could not be evaluated because data concerning antibiotic use were not available in our registry. Thus, further studies are needed to investigate whether prophylactic antibiotic use impacts functional outcome.

The incidence (14 %) of *sepsis during the advanced critical care period* in our study was relatively higher than that in previous studies [16, 18, 19]. A retrospective study showed a similar incidence (13 %), and septic events had no impact on neurological outcome at hospital discharge [43]. In our study, after adjusting for independent predictors of poor neurological outcome, sepsis, but not pneumonia, was significantly associated with poor neurological outcome. In a recent systematic review and meta-analysis of TTM induced in adults for any indication, TTM was strongly associated with an increased risk of pneumonia and sepsis [45]. Most studies and meta-analyses including cardiac arrest patients showed that pneumonia and sepsis may be more frequent after TTM but that there were no significant differences in mortality or neurological outcomes between subjects undergoing TTM and normothermia or maintenance at 36 °C [11, 16, 17]. In a prospective observational study including OHCA patients in Europe and North America, pneumonia and sepsis were inversely associated with mortality at 6 months based on univariate analysis [19]. The authors interpreted that the association of infections with mortality is possibly related to an increased risk of infection during a longer length of stay, which has been associated with a more favorable outcomes. However, pneumonia and sepsis were more frequent in patients with poor neurological outcome than in those with good neurological outcome in our study. The discrepancies between these two studies may be explained by the difference in the rate of WLST. The length of stay of the patients not awakening after sedation was typically longer than that of the patients who woke up after sedation was withdrawn because WLST was not performed in our study.

*Hypokalemia and hyperglycemia during the cooling period* were also frequently observed in our study, and these findings were consistent with those of previous reports [17–19]. Hypokalemia during the cooling period was not associated with poor neurological outcome, and this result was consistent with those of previous reports [17, 19]. Hyperglycemia was associated with an increased incidence of poor outcome in patients who were not treated with TTM [46–48]. However, the influence of the blood glucose level during TTM and the advanced critical care period is uncertain. Sustained hyperglycemia (>8 mmol/L or >144 mg/dL for >4 hours during the critical care period) was associated with increased mortality at 6 months in a prospective observational study of OHCA patients treated with TTM [19]. However, increased variability in the glucose level, but not the mean blood glucose level, during TTM was found to be an independent risk factor of in-hospital mortality in a prospective observational study [49]. In a recent large registry study that included OHCA patients treated with TTM, an increased median blood glucose level (>8.4 mmol/L or >152 mg/dL) over the first 48 hours was associated with poor neurological outcome [50]. In our study, hyperglycemia (>180 mg/dL) during the cooling period was associated with poor neurological outcome based on univariate analysis but not multivariate analysis. Our results suggest that a single recording of a high blood glucose level during the cooling period may not be an independent risk factor of poor neurological outcome.

Notably, the incidence of *hypoglycemia* (<4.4 mmol/L or <80 mg/dL) *within 72 hours after ROSC* was high (23 %) in our study and was significantly associated with poor neurological outcome at hospital discharge based on both univariate and multivariate analysis. These incidences were quite different from the results of the Target Temperature Management trial and an observational study (5.5 % and 5 %, respectively) in which hypoglycemia was defined at a lower level (<3.0 mmol/L or <54 mg/dl) than our definition [11, 19]. In the observational study, hypoglycemia during the critical care period was associated with increased mortality at 6 months based on univariate analysis but not multivariate analysis [19]. The reasons for the high incidence of hypoglycemia within 72 hours after ROSC in our study may be very complex. The lack of feeding, the use of intensive insulin therapy prior to the publication of the 2010 guidelines, and increased insulin sensitivity during rewarming may have influenced the high incidence of post-rewarming hypoglycemia within 72 hours after ROSC. Most hospitals that participated in our registry used no feeding protocol within 72 hours after ROSC because TTM may impair intestinal motility [15]. In a recent multicenter retrospective analysis, insulin sensitivity consistently increased over the first 36 hours [51]. Although the optimal level and duration of glycemic control during post-cardiac arrest care remains controversial, our results suggest that hypoglycemia within 72 hours after ROSC, even just below the normal range, may be harmful and may negatively impact neurological outcome because the injured brain is more sensitive to the glucose supply. Our results may support the recommendation of maintaining the blood glucose levels between 144 and 180 mg/dL based on the hypoglycemic risk of strict glucose control in the current guidelines [8, 9]. Our findings suggest that the careful monitoring and prevention of hypoglycemia are important within 72 hours after ROSC.

The incidences of *seizure and myoclonus during the advanced critical care period* were high in our study and were similar to those of previous reports, which ranged from 15 to 44 % [11, 19, 52, 53]. Seizure and myoclonus during the advanced critical care period were strongly associated with poor neurological outcome based on both univariate and multivariate analysis in our study, and these results were consistent with the results of previous studies [20, 54, 55]. Although anticonvulsant use was significantly associated with poor neurological outcome, this may not serve as an independent variable but rather may be collinear with seizure and myoclonus because most physicians in the participating hospitals in our registry only administered anticonvulsants when seizure or myoclonus was observed or highly suspected. The observed correlation between the use of anticonvulsants and poor outcome does not represent convincing evidence that this treatment contributes to poor outcome because patients with seizure after ROSC have likely experienced a major neurological injury.

Although seizure and myoclonus were strongly associated with poor neurological outcome, recent reports have suggested that some patients treated with TTM who experience seizure or myoclonus exhibit good neurological outcome [19, 56, 57]. In our study, 22 % of patients with myoclonus, 16 % of patients with seizure, and 25 % of patients treated with anticonvulsants exhibited good neurological outcome at the time of hospital discharge. These results were similar to the result that 17 % of OHCA patients with seizures exhibited good outcomes at 6 months in a previous observational study [19]. Therefore, careful monitoring and aggressive treatment of these AEs may be important for improving functional outcome.

An additional interesting finding in our study was the inverse association between *NMB use during TTM* and poor neurological outcome at hospital discharge. This result may support the hypothesis of Salciccioli et al. [58], who reported that early continuous NMB use for a 24-hour period is associated with an increased probability of survival among post-cardiac arrest patients. In their study, NMB use showed a trend toward improved functional outcome, although this result was not statistically significant. Although we did not consider the NMB type, NMB delivery protocol, or duration of NMB administration, our result generates the following hypotheses for future research: patients with poor neurological outcome need less NMB use for shivering control, and NMB use during the early post-cardiac arrest care period may improve functional outcome.

### Limitations

There were several limitations to our study. First, although we attempted to collect all consecutive OHCA patients who were treated with TTM in each hospital during the study period, there was an inevitable risk of selection bias because our study employed a retrospective design. Second, although we employed a standard investigation protocol, verified data completeness according to the site principle investigators, and used a team to perform data quality control, our study was subject to reporting bias because it was a registry-based multicenter study. Furthermore, the variability in TTM implementation timing and in the TTM protocol among hospitals may have impact the incidences of AEs and outcomes. Third, some of the AEs defined as other AEs, such as pneumonia and sepsis, may be considered cooling- or rewarming-related AEs. However, these AEs are difficult to diagnose during TTM and were recorded as other AEs during the advanced critical care period. Thus, our study cannot determine whether pneumonia or sepsis occurring during the cooling or rewarming period is associated with poor neurological outcome. Fourth, the data used in this study were collected from teaching hospitals in an Asian country; therefore, our results may have limited generalizability. Finally, we were only able to investigate neurological outcome at the time of hospital discharge, and this variable was not adequately blinded. This end point, which is not the current standard, may be not a reliable indicator of long-term outcome, although some investigators have suggested that the CPC score at the time of hospital discharge is a useful surrogate measure of long-term outcome [59].

## Conclusions

In this retrospective study using multicenter registry data, cooling- and rewarming-related AEs were not associated with poor neurological outcome at the time of hospital discharge in OHCA patients treated with TTM. However, sepsis, myoclonus, seizure, hypoglycemia within 72 hours after ROSC, and the use of anticonvulsants during the advanced critical care period were significantly associated with poor neurological outcome at the time of hospital discharge in our study.

## Key messages

- In this multicenter, registry-based study of adult OHCA patients treated with TTM in whom WLST was not performed, older age, previous poor health, a non-shockable initial rhythm, a longer time from arrest to ROSC, a non-cardiac cause of arrest, and CRRT were independent predictors of poor neurological outcome at the time of hospital discharge. Alternatively, initial GCS score ≥5, initial presence of the pupillary light reflex, and coronary angiography were inversely associated with poor neurological outcome.
- After adjusting for independent predictors of poor neurological outcome, cooling- and rewarming-related AEs were not significantly associated with poor neurological outcome at the time of hospital discharge.
- After adjusting for independent predictors of poor neurological outcome, sepsis, myoclonus, seizure, hypoglycemia within 72 hours after ROSC, and the use of anticonvulsants during the advanced critical care period were significantly associated with poor neurological outcome at the time of hospital discharge.

## Additional files

Additional file 1:
**KORHN registry case report form.** Korean Hypothermia Network (KORHN) registry case report form. Additional file 2:
**KORHN registry investigator manual.** Korean Hypothermia Network (KORHN) registry investigator manual. Additional file 3: Figure S1. Definition of the period in post-cardiac arrest care. **Figure S2.** Geographic distribution and the number of patients enrolled in the 24 participating centers. Additional file 4:
**Sensitivity analysis II.** Results of the sensitivity analysis for the patients who survived after sedation was withdrawn. Additional file 5:
**The name of ethics bodies and site principal investigators in the participating hospitals.**

## Abbreviations

95 % CI: 95 % confidence interval
AE: adverse event
AUC: area under the receiver operating characteristic curve
CABG: coronary artery bypass graft
CPC: cerebral performance category
CPR: cardiopulmonary resuscitation
CRRT: continuous renal replacement therapy
ECMO: extracorporeal membrane oxygenation
ED: emergency department
EEG: electroencephalography
EMS: emergency medical services
GCS: Glasgow coma scale
IABP: intra-aortic balloon pump
ICU: intensive care unit
MAP: mean arterial pressure
NMB: neuromuscular blocker
OHCA: out-of-hospital cardiac arrest
OR: odds ratio
PCI: percutaneous coronary intervention
ROSC: return of spontaneous circulation
SBP: systolic blood pressure
STEMI: ST-segment elevation myocardial infarction
TTM: targeted temperature management
WLST: withdrawal of life-sustaining treatment
