# Supplementary material for: Adverse events associated with poor neurological outcome during targeted temperature management and advanced critical care after out-of-hospital cardiac arrest
Source: Crit Care. 2015 Jul 22;19(1):283. doi: 10.1186/s13054-015-0991-9 (PMC4511983; doi:10.1186/s13054-015-0991-9)
Supplement: Additional file 1: — KORHN registry case report form. Korean Hypothermia Network (KORHN) registry case report form. [file 13054_2015_991_MOESM1_ESM.pdf]

Additional file 1. Korean Hypothermia Network (KORHN) Registry Case Report Form

Recorder: _______________ Date of arrest: ________/ ____/____

Hospital Code		Type of ED Visit Direct visit □ Transferred □	
Initial of Name	Age yyyy-mm-dd	Sex male □ female □	Best CPC before arrest ( )
Premorbid status	Previous healthy □ previous cardiac arrest □ myocardial infarction □ angina pectoris □ CHF (NYHA) □ HTN □ DM □ pulmonary disease stroke □ □ neurological disease □ renal dz. □ LC □ malignancy □		
**Resuscitation variables**			
Pre-EMS	Witness yes □ no □	Bystander CPR yes □ no □	
EMS	Initial rhythm VF/VT □ PEA □ asystole □ Unknown shockable □ Unknown non-shockable □ Unknown □		Chest compression yes □ no □
	Airway No device □ BVM □ Advanced airway □	AED yes □ no □	Number of shocks ( )
ED	Initial rhythm VF/VT □ PEA □ asystole□	Number of shocks ( )	Epinephrine ___mg
Time factor	Witnessed (yyyy-mm-dd-hh-mm), Encountered (if not witnessed) (yyyy-mm-dd-hh-mm), ROSC (yyyy-mm-dd-hh-mm)		
Cause of arrest	Cardiac □ submersion □ drug overdose or side effects □ asphyxia □ exsanguination □ hanging □ other non-cardiac □		
**Post-resuscitation variables**			
Breathing	Spontaneous breathing after ROSC yes □ no □ 24hr yes □ no □ 48hr yes □ no □ 72hr yes □ no □		
	Chest X-ray (0-1h)	Pneumothorax yes □ no □ Hemothorax yes □ no □ Pulmonary edema yes □ no □ Pulmonary contusion yes □ no □ Evidence of aspiration (0-1h) yes □ no □	
Circulation	Rate 0-1h (/) 1-24h (/) 24-48h (/) 48-72h (/)		
	Lactate (0-1h) ( )		
	STEMI yes □ no	Clinical evidence of cardiogenic shock yes □ no □	
	ECMO/IABP/CRRT yes□/□/□ no □/□/□ If yes, record the time (yyyy-mm-dd-hh-mm)		
	ICD/Pacemaker yes□/□ no □/□ If yes, record the time (yyyy-mm-dd-hh-mm)		
	Holter/EP yes □/□ no □/□ If yes, record the time (yyyy-mm-dd-hh-mm)		
	Thrombolysis/Angiography/PCI/CABG/Anticoagulant/Antiplatelet yes □/□/□/□/□/□ no □/□/□/□/□/□ If yes, record the time (yyyy-mm-dd-hh-mm)		
Neurological	After ROSC or arrival	GCS (E___V___M___) Pupillary light reflexes yes □ no □	Corneal reflex yes □ no □
	Involuntary movement	Seizure yes □ no □ Myoclonus yes □ no □	
	EEG	yes □ no □ If yes, record the time (yyyy-mm-dd-hh-mm) and findings ( )	
	SSEP N20 peak	yes □ no □ If yes, record the time (yyyy-mm-dd-hh-mm)	
		bilateral absent □ unilateral absent □ bilateral present □	
	Brain imaging	Brain CT yes □ no □ date and time (yyyy-mm-dd-hh-mm)	
		MRI yes □ no □ type ( ) date and time (yyyy-mm-dd-hh-mm)	
		Other study ( ) date and time (yyyy-mm-dd-hh-mm)	
	Anticonvulsants	yes □ no □ type ( )	
	Analgesia	yes □ no □ duration ( )	
	Sedation	yes □ no □ duration ( )	
	Paralysis	yes □ no □ duration ( )	
Metabolic	Glucose (0-1h) ( ) Insulin treatment yes □ no □ Hypoglycemia (<72hrs, <80mg/dl) yes □ no □		
	Attempted enteral feeding yes □ no □ Attempted parenteral feeding yes □ no □ If yes, time (yyyy-mm-dd-hh-mm)		
Therapeutic hypothermia	Target temperature (℃)		
	Induction start (yyyy-mm-dd-hh-mm) Maintenance start (yyyy-mm-dd-hh-mm) Rewarming start (yyyy-mm-dd-hh-mm) Reach to normal temperature (yyyy-mm-dd-hh-mm)		
	Induction technique	Cooling blanket or mattress □ Ice bag □ Cold saline or lactate Ringer □ Endovascular catheter □ Cold lavage or irrigation □ Linen □ Fan □ Hydrogel pad □ Cooling garment □ Extracorporeal circulation □ Others ( )	
	Maintenance technique	Cooling blanket or mattress □ Ice bag □ Cold saline or lactate Ringer □ Endovascular catheter □ Cold lavage or irrigation □ Linen □ Fan □ Hydrogel pad □ Cooling garment □ Extracorporeal circulation □ Others ( )	
	Rewarming technique	Cooling blanket or mattress □ Ice bag □ Cold saline or lactate Ringer □ Endovascular catheter □ Cold lavage or irrigation □ Linen □ Fan □ Hydrogel pad □ Cooling garment □ Extracorporeal circulation □ Others ( )	
	Temperature monitoring site	Rectum □ Tympanic □ Esophagus □ Bladder □ Pulmonary artery □ Brain □ Others ( )	
	Reasons to stop cooling	Refractory shock yes □ no □ Severe arrhythmia yes □ no □ Severe bleeding yes □ no □ DNAR yes □ no □ Others ( )	
Adverse Events	Cooling-related	Overcooling yes □ no □ Bradycardia yes □ no □ If yes, need to treatment yes □ no □ Tachyarrhythmia yes □ no □ Hypokalemia yes □ no □ Hyperglycemia yes □ no □ Bleeding yes □ no □ If yes, need to transfusion yes □ no □ Hypotension yes □ no □	
	Rewarming-related	Rebound hyperthermia yes □ no □ Arrhythmia yes □ no □ Hyperkalemia yes □ no □ Hypoglycemia yes □ no □ Bleeding yes □ no □ If yes, need to transfusion yes □ no □ Hypotension yes □ no □	
	Other adverse events	Sepsis yes □ no□ (severe sepsis yes □ no □, septic shock yes □ no □) Pneumonia yes □ no □	
**Outcome variables**			
ICU admission yes □ no □	Survival discharge yes □ no □	CPC at hospital discharge ( )	
Cause of death	Hypoxic brain injury □ Multiple organ failure □ Sepsis □ Others □ ( )	Hospital death date (HD#)	
