# Supplementary material for: Adverse events associated with poor neurological outcome during targeted temperature management and advanced critical care after out-of-hospital cardiac arrest
Source: Crit Care. 2015 Jul 22;19(1):283. doi: 10.1186/s13054-015-0991-9 (PMC4511983; doi:10.1186/s13054-015-0991-9)
Supplement: Additional file 2: — KORHN registry investigator manual. Korean Hypothermia Network (KORHN) registry investigator manual. [file 13054_2015_991_MOESM2_ESM.pdf]

Additional file 2. Korean Hypothermia Network (KORHN) Registry Investigator Manual

Data field	Definition
**Patient enrolment information**	
Recorder	Record the name of investigator who registered the patient data
Date of arrest	Record the date of arrest in yyyy-mm-dd format
Hospital code	Three letters of alphabetical codes are predefined hospital codes for each hospital and following 4 serial numbers are automatically generated in the system
**Patient demographics**	
Type of ED Visit	Direct visit or Transferred
Initial of Name	English initial of the patients' name
Age	Record the birthday in yyyy-mm-dd format
Sex	Select male or female.
Best CPC before arrest	Select the cerebral performance category (CPC) score<br>CPC1 – Good cerebral performance, able to work and normal life<br>CPC2 – Moderate cerebral disability, disabled but independent<br>CPC3 – Severe cerebral disability, conscious but disabled and dependent<br>CPC4 – Coma/vegetative state, unconsciousness
Premorbid status	Multiple selections are allowed.<br>Previous healthy define absence of the listed diseases
**Resuscitation variables**	
**1.Pre-EMS**	
Witnessed	A cardiac arrest that is seen or heard by another person or is monitored
Bystander CPR	Cardiopulmonary resuscitation performed by a person who is not responding as part of an organized emergency response system to a cardiac arrest
**2.EMS**	
Initial rhythm	Select the one monitored rhythm.<br>Unknown shockable - shock on AED which is not monitored the rhythm<br>Unknown unshockable - no shock on AED which is not monitored the rhythm<br>Unknown - no information is available
Chest compression	Select yes or no
Airway	Select one method. Advanced airway was defined endotracheal intubation or supraglottic devices
AED	Any AED apply with/without shock
Number of shocks	Number of shock performed by EMT
**3.Emergency department**	
Initial rhythm	Select the one monitored rhythm
Number of shocks	Number of shock performed by ED personnel
Epinephrine	Total dose
**4.Time factors**	
Witnessed time	Record witnessed time in yyyy-mm-dd-hh-mm format
Encountered time	If not witnessed, record encountered time in yyyy-mm-dd-hh-mm format
ROSC	A palpable pulse or generating a blood pressure was sustained for >20 min (or return of circulation if extracorporeal circulatory support is applied).
Cause of arrest	Select the one cause<br>Other non-cardiac includes anaphylaxis, excessive cold, excessive heat, hanging, lightning, electrocution, terminal illness, venomous sting, chemical poisoning, radiation exposure, etc.
**Post-resuscitation variables**	
**1.Breathing**	
Spontaneous breathing	Select yes if presence of spontaneous breathing at each time point
Chest X-ray (0-1 h)	Select yes if presence of each finding on chest X-ray checked within 1 hour after ROSC
**2.Circulation**	
Rate	Record highest and lowest values at each time point
Lactate (0-1 h)	Record the value within 1 hour after ROSC
Clinical evidence of cardiogenic shock	Hypotension (SBP <90 mmHg or MAP <60mmHg for at least 30 min or the need for supportive measures (fluid loading and vasopressors or inotropic or both) to maintain a SBP > 90 mmHg or MAP >60 mmHg) and end-organ hypoperfusion (cool extremities)
Cardiac interventions	Select yes if the intervention was performed and record time in yyyy-mm-dd-hh-mm format
**3.Neurological**	
Glasgow coma scale	Record each value after ROSC or ED arrival in transferred case
Pupillary light reflexes	Select yes if presence after ROSC or ED arrival in transferred case
Corneal reflexes	Select yes if presence after ROSC or ED arrival in transferred case
Seizure	Involuntary movement or no movement with epileptiform discharge on EEG

Myoclonus	Involuntary movement without epileptiform discharge on EEG
EEG	Select yes if EEG was performed and record time in yyyy-mm-dd-hh-mm format
SSEP N20 peak	Select yes if SSEP was performed and record findings and time in yyyy-mm-dd-hh-mm format
Brain imaging	Select yes if the imaging study was performed and record time in yyyy-mm-dd-hh-mm format
Anticonvulsants	Select yes if anticonvulsants were used and record names of the drugs
Analgesia	Select yes if analgesic was used and record the duration
Sedation	Select yes if sedative was used and record the duration
Paralysis	Select yes if neuromuscular blocker was used and record the duration
**4. Metabolic**	
Glucose (0-1h)	Record the highest value within 1 hour after ROSC
Insulin treatment	Select yes if insulin was used
Hypoglycemia	Select yes if any single event ≤80mg/dl which was developed within 72 h after ROSC
Attempted enteral feeding	Select yes if enteral feeding was started within 72 h after ROSC and record time in yyyy-mm-dd-hh-mm format
Attempted parenteral feeding	Select yes if parenteral feeding was started within 72 h after ROSC and record time in yyyy-mm-dd-hh-mm format
**5. Therapeutic hypothermia**	
Target temperature	Record target temperature level
Induction start	Record the time of cooling start in yyyy-mm-dd-hh-mm format
Maintenance start	Record the time for reaching at 34℃ in yyyy-mm-dd-hh-mm format
Rewarming start	Record the time of rewarming start in yyyy-mm-dd-hh-mm format
Reach to normal temperature	Record the time for reaching at 36.5℃ after rewarming in yyyy-mm-dd-hh-mm format
TH techniques	Select all type of devices which were used for the cooling and rewarming
Temperature monitoring site	Select all site which were used for the temperature monitoring
Reasons to stop cooling	Select the reason to stop cooling. If other reason was , describe the reason
**6. Adverse events**	
Overcooling	Drop of body temperature below 32℃ during induction and maintenance
Rebound hyperthermia	Increase of body temperature over 38℃ during rewarming
Bradycardia	Hear rate lower than 40 per minute
Tachyarrhythmia	Newly developed tachyarrhythmia except sinus tachycardia
Hypokalemia	Any single event ≤3.0mEq/L during the cooling induction and maintenance
Hyperkalemia	Any single event ≥5.0mEq/L during the rewarming
Hyperglycemia	Any single event ≥180mg/dl during the cooling induction and maintenance
Hypoglycemia	Any single event ≤80mg/dl during the rewarming
Bleeding	Any type of bleeding associated with TH
Hypotension	SBP <90 mmHg or MAP <60mmHg for at least 30 min or the need for supportive measures (fluid loading and vasopressors or inotropic or both) to maintain a SBP> 90 mmHg or MAP>60 mmHg
Other AEs	Adverse events developed within 7 days after ROSC
Sepsis	A clinical syndrome defined by the presence of both documented or suspected infection and a systemic inflammatory response syndrome
Severe sepsis	Sepsis with organ dysfunction, hypoperfusion or hypotension
Septic shock	Sepsis with hypotension (SBP<90mmHg or reduction of >40mmHg from baseline) or requirement for vasoactive agent
Pneumonia	Following 4 clinical criteria are required for diagnosis:<br>1) new or progressive consolidation on the chest radiograph, 2) fever, 3) leukocytosis, 4) presence of purulent tracheobronchial secretions
**Outcome variables**	
ICU admission	Select yes or no
Survival discharge	Select yes or no
CPC at hospital discharge	Select one CPC score at hospital discharge. The CPC score was abstracted from the medical charts or electronic medical records
Cause of death	Select the one cause<br>Hypoxic brain injury was defined as the death due to brain damage with no organ dysfunction<br>Multiple organ failure was defined as the death due to dysfunction of two more organs<br>Others include circulatory failure and other cause of arrest (e.g. rearrest due to sudden VF)
Hospital death date	Record hospital day after admission
