# Supplementary material for: Adverse events associated with poor neurological outcome during targeted temperature management and advanced critical care after out-of-hospital cardiac arrest
Source: Crit Care. 2015 Jul 22;19(1):283. doi: 10.1186/s13054-015-0991-9 (PMC4511983; doi:10.1186/s13054-015-0991-9)
Supplement: Additional file 3: Figure S1. — Definition of the period in post-cardiac arrest care. Figure S2. Geographic distribution and the number of patients enrolled in the 24 participating centers. [file 13054_2015_991_MOESM3_ESM.pdf]

Additional file 3. Supplementary figures

Figure 1. Definition of the period in post-cardiac arrest care

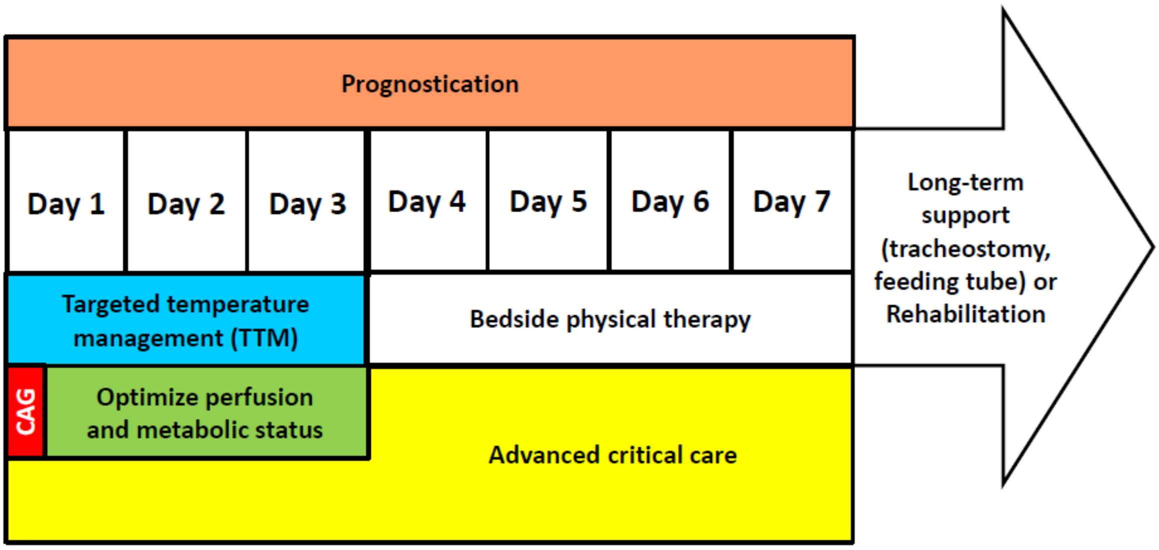

Figure 2. Geographic distribution and the number of patients enrolled in the 24 participating centers

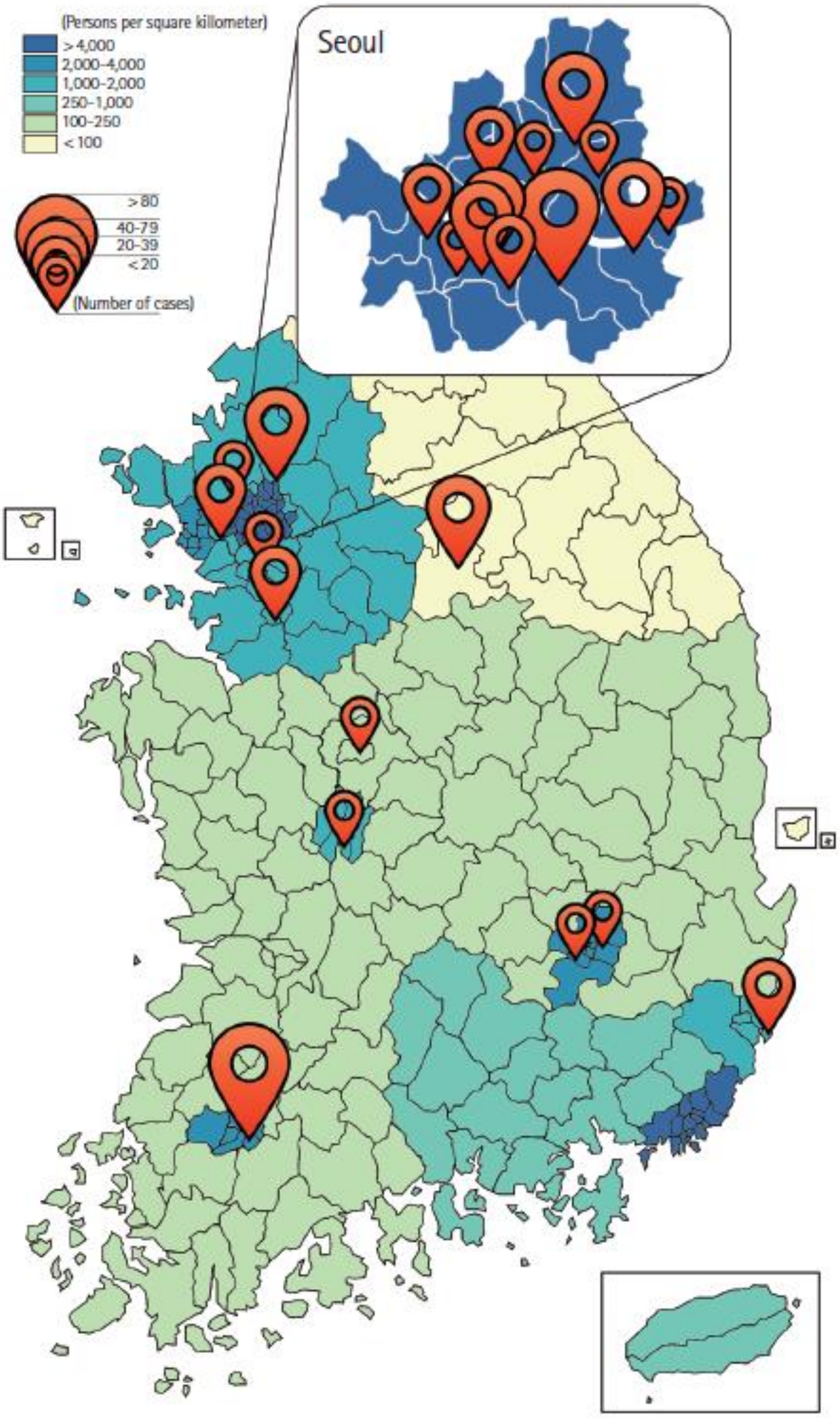

This figure was copied from the reference #21. (Byung Kook Lee, Kyu Nam Park, Gu Hyun Kang, et al. *Clin Exp Emerg Med* 2014, 1:19-27) with permission of the copylight owner (*Clin Exp Emerg Med*, eISSN2383-4625).
