# Supplementary material for: Adverse events associated with poor neurological outcome during targeted temperature management and advanced critical care after out-of-hospital cardiac arrest
Source: Crit Care. 2015 Jul 22;19(1):283. doi: 10.1186/s13054-015-0991-9 (PMC4511983; doi:10.1186/s13054-015-0991-9)
Supplement: Additional file 4: — Sensitivity analysis II. Results of the sensitivity analysis for the patients who survived after sedation was withdrawn. [file 13054_2015_991_MOESM4_ESM.pdf]

Additional file 4. Results of the sensitivity analysis for the patients who survived after sedation is withdrawn

Table 1. Baseline patient characteristics

Patient characteristics	Good neurological outcome (CPC 1 and 2) n=228 (33.9)	Poor neurological outcome (CPC 3-5) n=444 (66.1)	*p*
Background variables			
Age, years	50 (40 - 60)	59 (47 - 71)	<.001
Male	177 (77.6)	303 (68.2)	0.011
Previously healthy[a]	130 (57.0)	167 (37.6)	<.001
Myocardial infarction	13 (5.7)	22 (5.2)	0.776
Angina	19 (8.3)	29 (6.5)	0.391
Congestive heart failure	5 (2.2)	17 (3.8)	0.259
Hypertension	57 (25.0)	168 (37.8)	0.001
Diabetes mellitus	29 (12.7)	107 (24.1)	0.001
Pulmonary disease	4 (1.8)	32 (7.2)	0.003
Stroke	4 (1.8)	25 (5.6)	0.019
Other neurological disease	7 (3.1)	32 (7.2)	0.030
Renal disease	4 (1.8)	36 (8.1)	0.001
Liver cirrhosis	1 (0.4)	7 (1.6)	0.277
Malignancy	5 (2.2)	13 (2.9)	0.576
Resuscitation variables			
Witnessed arrest	182 (79.8)	273 (61.5)	<.001
Bystander CPR	85 (37.3)	111 (25.0)	0.001
First monitored rhythm			<.001
VF/ pulseless VT	148 (64.9)	84 (18.9)	
Pulseless electrical activity	28 (12.3)	75 (16.9)	
Asystole	41 (17.9)	274 (61.7)	
Unknown	11 (4.8)	11 (2.5)	
Cardiac cause of arrest	201 (88.2)	214 (48.2)	<.001
Time from arrest to ROSC, min	24.5 (16.5 - 34.5)	33 (25 - 45)	<.001
Post-resuscitation variables			
Initial Glasgow coma scale ≥5	76 (33.3)	21 (4.7)	<.001
Initial presence of pupillary light reflex	178 (78.1)	127 (28.6)	<.001
ST elevation myocardial infarction	36 (15.8)	22 (5.0)	<.001
Cardiogenic shock[b]	64 (28.1)	115 (25.9)	0.547
Thrombolysis	2 (0.9)	3 (0.7)	1.000
Coronary angiography	152 (66.7)	47 (10.6)	<.001
Percutaneous coronary intervention	44 (19.3)	22 (5.0)	<.001
Coronary artery bypass graft	7 (3.1)	0 (0.0)	<.001

Intra-aortic balloon pump	8 (3.5)	8 (1.8)	0.169
Extracorporeal membrane oxygenation	4 (1.8)	10 (2.3)	0.782
Continuous renal replacement therapy	4 (1.8)	45 (10.1)	<.001
Time from arrest to initiation of cooling, min	142 (81.5 - 247.5)	139.5 (78 - 232)	0.955
Time from arrest to 34℃, min	385 (257.5 - 570)	303.5 (196 - 454.5)	0.033
Rewarming time, min	666 (420 - 780)	720 (480 - 990.5)	0.001

CPC, cerebral performance category; CPR, cardiopulmonary resuscitation; VF, ventricular fibrillation; VT, ventricular tachycardia; ROSC, return of spontaneous circulation.
Data are presented as medians (interquartile range) or counts (percentage). [a]absence of the listed diseases, [b]hypotension (SBP <90 mmHg or MAP <60mmHg for at least 30 min or the need for supportive measures (fluid loading and vasopressors or inotropic or both) to maintain a SBP > 90 mmHg or MAP >60 mmHg) and end-organ hypoperfusion (cool extremities).

Table 2. Multivariable predictive model for poor neurological outcomes at the time of hospital discharge

Variables	OR	(95% CI)	*p*
Continuous renal replacement therapy[a]	6.02	(1.93-23.67)	0.004
Non-shockable initial rhythm[b]	3.20	(1.83-5.65)	<.001
Non-cardiac cause of arrest[c]	2.75	(1.47-5.27)	0.002
Previously unhealthy[d]	1.99	(1.14-3.50)	0.016
Time from arrest to ROSC[e]	1.06	(1.04-1.08)	<.001
Age[f]	1.03	(1.02-1.05)	<.001
Initial Glasgow Coma Scale ≥ 5[g]	0.30	(0.14-0.63)	0.002
Initial presence of pupillary light reflex[h]	0.28	(0.17-0.46)	<.001
Coronary angiography[i]	0.12	(0.07-0.21)	<.001

ROSC, return of spontaneous circulation; OR, odds ratio; 95% CI, 95% confidence interval
[a]no continuous renal replacement therapy as reference, [b]VF/pulseless VT as reference, [c]cardiac cause of arrest as reference, [d]absence of the listed diseases as reference, [e]by minute, [f]by year, [g]<5 as reference, [h]absence of pupillary light reflex as reference, [i]no coronary angiography as reference

Hosmer and Lemeshow Goodness-of-fit test	
Chi-square (DF)	7.4731 (8)
P value	0.4865
R square	0.4827
Max-rescaled R-square	0.6683

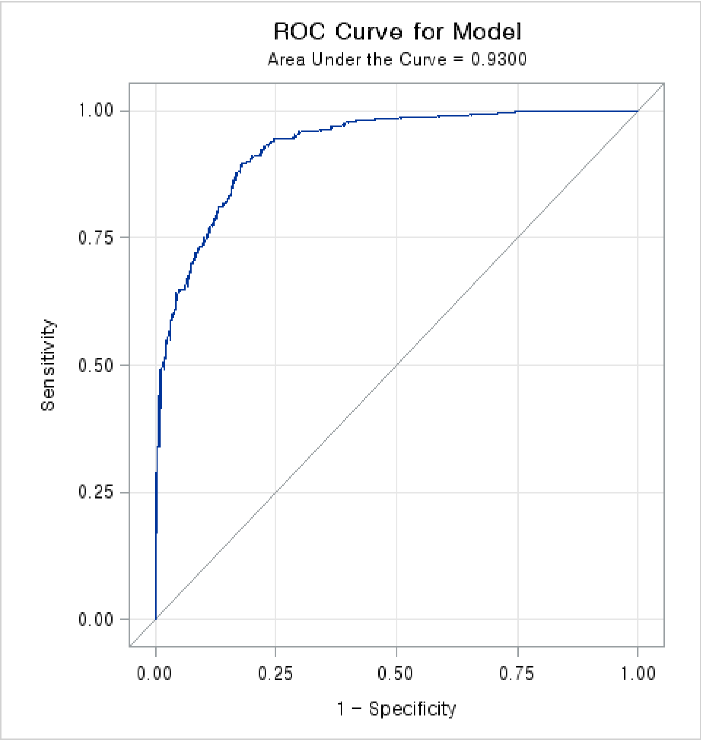

Table 3. Adverse events and concomitant treatments with corresponding odds ratios for poor neurological outcomes at the time of hospital discharge

Category	AEs and concomitant treatments	Total N (%)	CPC 1 and 2 n (%)	CPC 3-5 n (%)	OR (95% CI)	*p*	Adjusted OR (95% CI)	*p*
		672 (100)	228(32.4)	444 (67.6)				
Cooling -related AEs	Overcooling[a]	121 (18.0)	23 (10.1)	98 (22.1)	2.52 (1.55-4.10)	<.001	1.14 (0.56-2.31)	0.718
	Bradycardia[b]	79 (11.8)	35 (15.4)	44 (9.9)	0.61 (0.38-0.98)	0.040	0.73 (0.35-1.54)	0.408
	Tachyarrhythmia	60 (8.9)	24 (10.5)	36 (8.1)	0.75 (0.44-1.29)	0.299	0.54 (0.25-1.18)	0.124
	Hypokalemia[c]	208 (31.0)	65 (28.5)	143 (32.2)	1.19 (0.84-1.69)	0.326	1.16 (0.68-1.97)	0.599
	Hyperglycemia[d]	304 (45.2)	81 (35.5)	223 (50.2)	1.83 (1.32-2.54)	<.001	1.40 (0.85-2.30)	0.191
	Bleeding	20 (3.0)	6 (2.6)	14 (3.2)	1.21 (0.46-3.18)	0.707	0.93 (0.21-4.08)	0.920
	Hypotension[e]	190 (28.3)	54 (23.7)	136 (30.6)	1.42 (0.99-2.05)	0.059	1.01 (0.58-1.79)	0.961
Rewarming -related AEs	Rebound hyperthermia[f]	83 (12.4)	29 (12.7)	54 (12.2)	0.95 (0.59-1.54)	0.834	1.46 (0.66-3.23)	0.355
	Arrhythmia	39 (5.8)	10 (4.4)	29 (6.5)	1.52 (0.73-3.18)	0.263	1.42 (0.52-3.92)	0.496
	Hyperkalemia[g]	45 (6.7)	7 (3.1)	38 (8.6)	2.96 (1.30-6.73)	0.010	1.12 (0.37-3.33)	0.845
	Hypoglycemia[h]	68 (10.1)	22 (9.7)	46 (10.4)	1.08 (0.63-1.85)	0.772	1.19 (0.52-2.73)	0.686
	Bleeding	12 (1.8)	3 (1.3)	9 (2.0)	1.55 (0.42-6.79)	0.513	1.02 (0.16-6.44)	0.981
	Hypotension[e]	138 (20.5)	36 (15.8)	102 (23.0)	1.59 (1.05-2.42)	0.030	1.00 (0.54-1.87)	0.997
Other AEs during advanced critical care	Pneumonia[i]	271 (40.3)	70 (30.7)	201 (45.3)	1.87 (1.33-2.62)	<.001	1.00 (0.60-1.67)	0.995
	Sepsis	95 (14.1)	17 (7.5)	78 (17.6)	2.65 (1.52-4.59)	0.001	3.16 (1.38-7.22)	0.007
	Myoclonus[j]	150 (22.3)	34 (14.9)	116 (26.1)	2.02 (1.32-3.08)	0.001	3.71 (1.92-7.19)	<.001
	Seizure[k]	153 (22.8)	24 (10.5)	129 (29.1)	3.48 (2.18-5.57)	<.001	4.56 (2.29-9.10)	<.001
	Hypoglycemia[l]	156 (23.2)	36 (15.8)	120 (27.0)	1.98 (1.31-2.09)	0.001	2.13 (1.14-4.00)	0.019
Concomitant treatments	Anticonvulsants	281 (41.8)	71 (31.1)	210 (47.3)	1.98 (1.42-2.78)	<.001	1.75 (1.06-1.68)	0.030
	Insulin	436 (64.9)	136(59.7)	300 (67.6)	1.41 (1.01-1.96)	0.042	1.01 (0.61-1.68)	0.967
	Analgesics	314 (46.7)	122 (53.5)	192 (43.2)	0.66 (0.48-0.91)	0.012	0.77 (0.47-1.26)	0.298
	Sedatives	634 (94.4)	222 (97.4)	412 (92.8)	0.35 (0.14-0.85)	0.020	1.14 (0.33-3.97)	0.832
	Neuromuscular blockers	459 (68.3)	174 (76.3)	285 (64.2)	0.56 (0.39-0.80)	0.002	0.51 (0.29-0.89)	0.018

AEs, adverse events; CPC, cerebral performance category. [a]<32 ℃; [b]heart rate<40 bpm; [c]≤3.0 mEq/L; [d]≥180 mg/dL; [e] SBP<90 mmHg or MAP<60mmHg for at least 30 min or the need for supportive measures (fluid loading and vasopressors or inotropic or both) to maintain a SBP> 90 mmHg or MAP>60 mmHg; [f]>38 ℃ within 24 hours of the cessation of cooling; [g]≥5.0 mEq/L; [h]≤80 mg/dL within 72 h after ROSC; [i]new or progressive consolidation on the chest radiograph, fever, leukocytosis, and the presence of purulent tracheobronchial secretions; [j]clinically involuntary movement without epileptiform discharge on EEG; [k]either clinically involuntary movement with epileptiform discharge on electroencephalogram (EEG) or epileptiform discharge only on EEG; [l]≤80 mg/dL within 72 hours after ROSC
