# Supplementary material for: Adverse events associated with poor neurological outcome during targeted temperature management and advanced critical care after out-of-hospital cardiac arrest
Source: Crit Care. 2015 Jul 22;19(1):283. doi: 10.1186/s13054-015-0991-9 (PMC4511983; doi:10.1186/s13054-015-0991-9)
Supplement: Additional file 5: — The name of ethics bodies and site principal investigators in the participating hospitals. [file 13054_2015_991_MOESM5_ESM.docx]

| **City** | **Name of Ethical body** | **Principle Investigator** |
| --- | --- | --- |
| Anyang | Hallym University Sacred Heart Hospital IRB | Yoo Dong Son |
| Bucheon | Soonchunhyang University Hospital IRB | Hoon Lim |
| Cheongju | Chungbuk National University Hospital IRB | Jin Hong Min |
| Daegu | Kyungpook National University Hospital IRB | Jung Bae Park |
|  | Daegu Catholic University Medical Center IRB | Tae Chang Jang |
| Daejeon | Chungnam National University Hospital IRB | Yeon Ho You |
| Goyang | Inje University Ilsan Paik Hospital IRB | Kyung Hwan Kim |
| Gwangju | Chonnam National University Hospital IRB | Byung Kook Lee |
| Seoul | University of Ulsan Asan Medical Center IRB | Won Young Kim |
|  | Chung-Ang University Hospital IRB | Dong Hoon Lee |
|  | Ewha Womans University Mokdong Hospital IRB | Chul Han |
|  | Hallym University Kangdong Sacred Heart Hospital IRB | Gyu Chong Cho |
|  | Hallym University Kangnam Sacred Heart Hospital IRB | Gu Hyun Kang |
|  | Hanil General Hospital, Korea Electric Power Medical Corporation IRB | In Soo Cho |
|  | Kyung Hee University Medical Center IRB | Jong Seok Lee |
|  | Seoul National University Borame Medical Center IRB | Jonghwan Shin |
|  | Seoul National University Hospital IRB | Gil Joon Suh |
|  | Catholic Medical Center / Seoul St. Mary’s Hospital IRB | Kyu Nam Park |
|  | Yonsei University Severance Hosptial IRB | Yoo Seok Park |
|  | Catholic Medical Center / Yeouido St. Mary’s Hospital IRB | Seung Pill Choi |
| Suwon | Ajou University Hospital IRB | Gi Woon Kim |
| Uijeongbu | Catholic Medical Center / Uijeongbu St. Mary’s Hospital IRB | Joo Suk Oh |
| Ulsan | Ulsan University Hospital IRB | Wook Jin Choi |
| Wonju | Yonsei University Wonju Severance Christian Hospital IRB | Kyoung Chul Cha |
